# Peer Coaching Through mHealth Targeting Physical Activity in People With Parkinson Disease: Feasibility Study

**DOI:** 10.2196/mhealth.8074

**Published:** 2018-02-15

**Authors:** Cristina Colón-Semenza, Nancy K Latham, Lisa M Quintiliani, Terry D Ellis

**Affiliations:** ^1^ Center for Neurorehabilitation, Department of Physical Therapy & Athletic Training College of Health & Rehabilitation Sciences: Sargent College Boston University Boston, MA United States; ^2^ Boston Claude D Pepper Older Americans Independence Center Research Program in Men’s Health: Aging and Metabolism Brigham and Women’s Hospital, Harvard Medical School Boston, MA United States; ^3^ School of Medicine Department of Medicine, Section of General Internal Medicine Boston University Boston, MA United States

**Keywords:** Parkinson disease, exercise, telemedicine, social support, fitness tracker

## Abstract

**Background:**

Long-term engagement in exercise and physical activity mitigates the progression of disability and increases quality of life in people with Parkinson disease (PD). Despite this, the vast majority of individuals with PD are sedentary. There is a critical need for a feasible, safe, acceptable, and effective method to assist those with PD to engage in active lifestyles. Peer coaching through mobile health (mHealth) may be a viable approach.

**Objective:**

The purpose of this study was to develop a PD-specific peer coach training program and a remote peer-mentored walking program using mHealth technology with the goal of increasing physical activity in persons with PD. We set out to examine the feasibility, safety, and acceptability of the programs along with preliminary evidence of individual-level changes in walking activity, self-efficacy, and disability in the peer mentees.

**Methods:**

A peer coach training program and a remote peer-mentored walking program using mHealth was developed and tested in 10 individuals with PD. We matched physically active persons with PD (peer coaches) with sedentary persons with PD (peer mentees), resulting in 5 dyads. Using both Web-based and in-person delivery methods, we trained the peer coaches in basic knowledge of PD, exercise, active listening, and motivational interviewing. Peer coaches and mentees wore FitBit Zip activity trackers and participated in daily walking over 8 weeks. Peer dyads interacted daily via the FitBit *friends* mobile app and weekly via telephone calls. Feasibility was determined by examining recruitment, participation, and retention rates. Safety was assessed by monitoring adverse events during the study period. Acceptability was assessed via satisfaction surveys. Individual-level changes in physical activity were examined relative to clinically important differences.

**Results:**

Four out of the 5 peer pairs used the FitBit activity tracker and *friends* function without difficulty. A total of 4 of the 5 pairs completed the 8 weekly phone conversations. There were no adverse events over the course of the study. All peer coaches were “satisfied” or “very satisfied” with the training program, and all participants were “satisfied” or “very satisfied” with the peer-mentored walking program. All participants would recommend this program to others with PD. Increases in average steps per day exceeding the clinically important difference occurred in 4 out of the 5 mentees.

**Conclusions:**

Remote peer coaching using mHealth is feasible, safe, and acceptable for persons with PD. Peer coaching using mHealth technology may be a viable method to increase physical activity in individuals with PD. Larger controlled trials are necessary to examine the effectiveness of this approach.

## Introduction

### Background

For persons with Parkinson disease (PD), exercise and physical activity reduce impairments, improve function, enhance quality of life, and potentially modify disease progression [1-4]. Despite this evidence and recommendations by neurologists to exercise [5], most individuals with PD are physically inactive [6]. Walking, a highly accessible form of physical activity, has been shown to decline early in the course of the disease and therefore is an important target of intervention [7,8]. Results of exercise trials in PD reveal the benefits of moderate-intensity walking to reduce disability [9,10]. Although the optimal dose of moderate-intensity exercise in PD is not known, exercise guidelines published by the American College of Sports Medicine [11] for older adults are routinely applied to persons with PD [12,13]. Recommendations consist of 150 min of moderate-intensity exercise per week, the equivalent of approximately 30 min, 5 days per week [11,14]. Studies in PD reveal a pattern of sedentary behavior with 73% failing to reach this recommendation [15]. Studies that have successfully engaged participants with PD in exercise have typically done so under highly controlled conditions, in a clinical setting, under the direct supervision of a health care professional [10,13,16]. However, it is often not feasible or cost-effective for health care professionals to administer exercise programs on an ongoing basis, and clinic-based programs present many logistical barriers over the long term (ie, time constraints, transportation) [17].

A sustainable, scalable approach to increasing participation in long-term physical activity is needed to reduce disability in people with PD. We propose that training peers as coaches, using mobile health (mHealth) technology to facilitate remote interactions, may be a viable approach to help motivate people with PD to participate in exercise over the long term [18]. Peer coaching is a form of support in which peers with the same condition share disease-specific information, strategies for implementing lifestyle changes, and provide psychosocial support to overcome challenges associated with living with a particular condition [19,20]. Peer coaches who successfully participate in regular exercise could support sedentary peers to increase physical activity through cooperative goal setting, modeling the desired behavior, and providing regular feedback toward goals via shared mHealth platforms [20,21].

A growing knowledge base supports the use of peer coaching for people with chronic health conditions [20,21]. For example, those who underwent coronary artery bypass graft surgery experienced increased physical activity and self-efficacy with peer coaching [22]. Studies suggest that peer-led interventions in older adults and in individuals with type 2 diabetes were just as effective in increasing physical activity as professionally delivered interventions [23,24]. A significantly greater effect for long-term maintenance of physical activity (including walking) was found for a peer-led physical activity intervention compared with a control group that received pedometers and access to an exercise facility [25]. In a systematic review of peer-delivered physical activity interventions, increases in physical activity with peer mentoring were greater than those of an attention-matched control group and a no-intervention control group [19].

No theoretically based peer-led program for increasing physical activity currently exists for people with PD. A training program for peer mentors is needed to provide people with PD the skills, knowledge, and support needed to begin this new role [23]. Mentoring people with progressive neurological diseases, such as PD, to increase their physical activity presents several challenges, such as addressing problems with motor skill loss, the nonmotor symptoms such as apathy, as well as the progressive nature of the disease.

Higher self-efficacy for exercise among people with PD has been associated with successful participation in physical activity and therefore may be an important target of treatment [26]. Vicarious experiences, goal setting, and the provision of regular feedback have all been identified as important elements in increasing self-efficacy for exercise [26,27]. Integrating mHealth technology into the peer-mentoring approach could provide a means of incorporating the critical self-efficacy elements into daily life. Previous peer-mentored interventions have used pedometers to increase physical activity; however, the use of an activity tracker that also allows for real-time sharing of accumulated walking data (via FitBit *friends*) provides a more robust mechanism to increase self-efficacy. Using an activity tracker (FitBit) and becoming FitBit *friends* allows for remote interaction while simultaneously providing a medium for vicarious experiences, social comparison, and daily feedback on walking goals.

### Objectives

The purpose of this study was to develop a PD-specific peer coach training program and a remote peer-mentored walking program using mHealth technology with the goal of increasing physical activity in persons with PD. Moreover, we set out to examine the feasibility, safety, and acceptability of the programs along with preliminary evidence of individual-level changes in walking activity, self-efficacy, and disability in the peer mentees.

## Methods

### Development of the Peer Coach Training Program (Peer Coaches Only)

#### Theoretical Framework

A peer coach training program was developed by the authors based on the self-determination theory and Bandura’s social cognitive theory [28,29]. The self-determination theory proposes that autonomy (supported through individualized goals in partnership with coach and by enhancing empowerment), competence (supported through coach focusing on acceptance and affirmations of previous and ongoing successes and strengths with physical activity), and relatedness (supported through FitBit *friends* and weekly phone conversations) drive motivation for behavior. The social cognitive theory is focused on building self-efficacy through social structures and experiences to drive behavior change. Peer mentoring, with the addition of regular mHealth interactions, may increase social comparison and enhance self-efficacy, leading to the adoption of increased physical activity. The program incorporated key elements from other successful peer coach training programs [20,22,23,30] as well as content that was identified as being important to persons with PD.

#### Training Program

Before the in-person training, peer coaches were asked to review printed and Web-based educational materials independently over a 1- to 2-week period in their homes at a self-selected pace (approximately 3-4 hours). Educational materials were provided in both hard copy (printed material, handbooks) and on a flash drive with links to websites that provided an overview of PD, the benefits of exercise, strategies to improve motivation, and the benefits of social support as well as an introduction to the activity tracker and peer support [16,31-34]. Information on ethics, roles, and responsibilities of being a peer mentor, and community resources were also provided. Next, peer coaches participated in two, 4-hour, in-person training sessions, separated by 1 week, at the Center for Neurorehabilitation at Boston University. The training program was administered by a physical therapist who was board certified in neurology (CCS). The topics included motivational interviewing, active listening, action plans, and instruction on the technology used in this study and were presented through lectures, discussions, and role-playing (Textbox 1). Case examples related to living with PD were used to integrate these concepts and strengthen skill acquisition.

### Study Design and Participants

**Figure 1:**
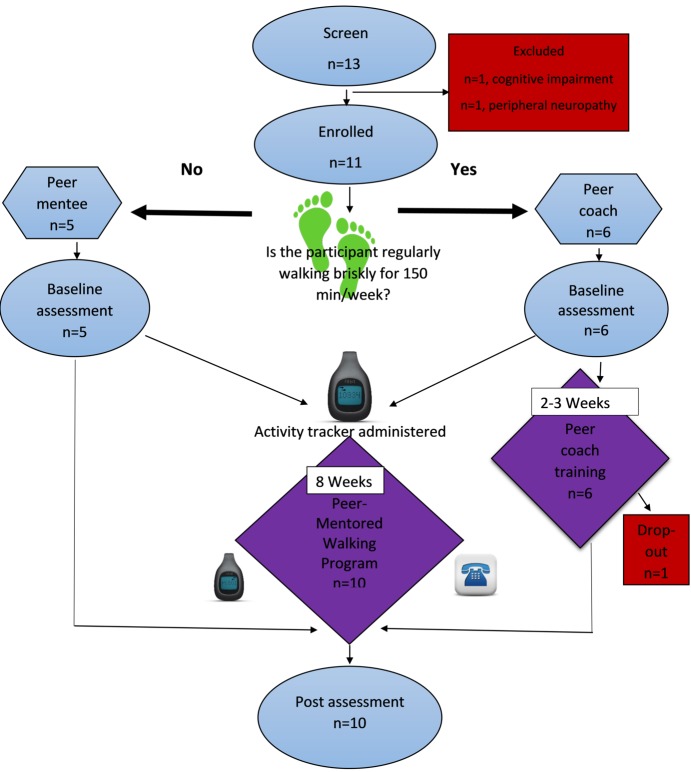
Participant flowchart.

Trained peer coaches were matched with peer mentees of the same sex based on previous successful peer support programs that matched peer pairs by sex [20,22,35]. Each peer dyad participated in the walking program. All outcomes were assessed at baseline and post intervention with the exception of walking activity, which was measured with the activity tracker at baseline and then during the final 7 consecutive days of activity tracking (Figure 1).

Adults with idiopathic PD were recruited through a patient registry at the Center for Neurorehabilitation at Boston University and postings in the newsletter of the American Parkinson Disease Association, MA Chapter, Information and Referral Center. Interested individuals were screened in person for eligibility. Inclusion criteria included a diagnosis of idiopathic PD (using UK Brain Bank Criteria), Hoehn and Yahr stage of 1-3, Montreal Cognitive Assessment (MOCA) >24, a stable dose of Parkinson’s medications for at least 2 weeks before study onset, able to walk without physical assistance or an assistive device for at least 10 continuous minutes, and able to effectively communicate with recruitment personnel. Exclusion criteria were a diagnosis of atypical Parkinsonism, more than 2 falls in the previous month (due to safety reasons), a score of 3 or greater on item number 3 of the Freezing of Gait questionnaire (often or always freezing with walking), and serious comorbidities (ie, heart failure, diabetes mellitus, or cancer) that may interfere with the ability to participate in a walking program. Trained research assistants, who were not involved in the intervention, completed the assessments. Those participants who were meeting or exceeding national exercise guidelines [11,14] by engaging in brisk walking greater than or equal to 150 min per week, measured by self-report, before study onset, were designated as peer coaches. Those who were not walking or walking below this level, before study onset, were designated as peer mentees. This study was approved by the Institutional Review Board at Boston University. Informed consent was obtained from all study participants.

### Peer-Mentored Walking Program

#### Initial Setup

The peer coaches and mentees were given a wireless activity tracker (FitBit Zip) and were instructed on how to use the device during their initial visit to the Center for Neurorehabilitation at Boston University. Participants were instructed on how to view their daily accumulated steps on the activity tracker screen. They were assisted with syncing this tracker with their device(s) (smartphone, tablet, or laptop). Coaches were instructed on how to become *friends* on the Fitbit mobile app, so they were prepared to instruct mentees during their initial interaction.

#### Walking Goal, Action Plan, and mHealth Interactions

The peer coach contacted the peer mentee, either by phone or email, within 1 week of completing the peer coach training to schedule an initial conversation. This initial conversation focused on establishing rapport, jointly determining the 8-week walking goal for the mentee, and developing the initial action plan. The walking goals for mentees were increased from the step averages obtained via the activity tracker during the baseline period. There was no predetermined increase for the walking goal, as this was individualized and based on the peer coach and peer mentee’s mutually agreed-upon goal and action plan. The peer *coach* did not have an explicit step goal. The action plan specified the location, days of the week, time of the day, duration, and with whom the peer mentee would engage in walking activity. The peer coach and peer mentee did not walk together, and instead, they each walked in their own self-selected environment. The action plan also included an assessment of the participant’s confidence in their ability to reach their goal. If their confidence to achieve the goal was lower than 80%, the goal was revised until their confidence in achieving the goal was elevated to 80% or greater. The peer coach also instructed the peer mentee on how to become FitBit *friends* during the initial interaction and explained how they could assess the walking goal and view each other’s steps remotely. Peer pairs viewed the steps they accumulated over the week using the FitBit *friends* option. The FitBit *friends* feature allows for remote interaction between the peer coach and peer mentee, providing an opportunity for regular feedback (ie, cheering with an emoji or instant messaging) on progress toward goals. Mentees could see the coach’s step counts, providing a social comparison and vicarious experiences leading to greater self-efficacy among mentees.

**Peer coach training program: in-person skill-based learning components.**

Motivational interviewing

- Spirit
  - Partnership
  - Empowerment
  - Acceptance
  - Compassion
  - Evocation

- Skills
  - Open-ended questions
  - Affirmations
  - Reflections
  - Summaries

- Processes
  - Engaging
  - Focusing
  - Evoking
  - Planning

- Goals
  - Specific
  - Measurable
  - Achievable
  - Relevant
  - Timed

Active listening

- Building rapport
- Enhancing understanding
- Establishing trust

Technology

- FitBit management
  - Donning
  - Changing batteries
  - Syncing with personal device
  - Using the app
  - Using the *friends* function

Action plans

- Self-management
- Assist with specifics (day, time, location, duration, with whom)
- Identifying barriers
- Dealing with conflict

#### Weekly Phone Calls

The peer coach and mentee engaged in phone conversations weekly over the 8-week study period from the convenience of their own homes. Peer coaches were given paper calendars for scheduling weekly phone calls and guiding checklists to guide peer discussions to ensure that they were adhering to the recommended techniques for peer mentoring. Peers discussed the following elements on the guiding checklist: assessing the walking activity goal of the peer mentee, progress made, problems encountered, strategies to overcome barriers, and resources available. They jointly solved the problem about how to increase participation in walking activity within daily life at home and in the community.

Guidance and support was provided by the physical therapist on the research team to the peer coaches via conference calls, following the initial and 4-week mentor-mentee conversations. This included reinforcing the role of the peer coach, ensuring successful use of the activity tracker, and strategies to assist coaches with potential challenges encountered when mentoring the peer mentee.

### Outcomes

#### Feasibility

Feasibility was determined by examining recruitment, participation, and retention rates. Recruitment was assessed by examining recruitment rates and the sample characteristics of those recruited. Participation was assessed by monitoring the completion of phone calls between the peer coach and peer mentee, which were recorded by the peer coaches on a calendar (63%, 5/8 calls set as criteria), and use of the mHealth platform (80%, 4/5 peer pairs as criteria). Retention was assessed by tracking the number of participants who completed the peer coach training program (coaches only) and mentored walking program (coaches and mentees) (80%, [5/6] retention for peer coach training program and 80% [9/11] retention for mentored walking program set as criteria).

#### Safety

Adverse events were monitored throughout the study period. Participants were instructed to contact a research assistant if there were any falls or a change in status that led to medical attention. Contacts were to be recorded in a database by the research assistant.

#### Acceptability

At the final assessment, peer coaches responded to 12 questions about their satisfaction with the training program and 7 questions about their perception of the effectiveness of the training program. Acceptability criteria were set as 80% (4/5) of participants were satisfied to very satisfied, agreed that the training was clear , and had confidence in their ability to coach after the training . A 1-hour focus group was conducted with all peer coaches 1 week after the last peer interaction to discuss successes, challenges, and reactions to the peer coaching experience. A research assistant took detailed notes throughout the session. Peer mentees responded to 13 questions about their satisfaction with the peer-mentored walking program (80% satisfied to very satisfied, endorsed the peer interaction was enjoyable, and built confidence to manage physical activity were set as criteria).

#### Walking Activity

Using the activity tracker, walking was measured as average steps per day for 7 days, active minutes per week, and the frequency of achieving 30 min of fairly active to very active minutes over 7 days before the peer-mentored walking program began and again over the last 7 consecutive days in which the activity tracker was worn. Research assistants downloaded all activity data during the participants’ last study visit.

#### Self-Efficacy

Self-efficacy was measured using the Self-Efficacy for Walking-Duration, a 10-item questionnaire that assesses self-efficacy for walking moderately fast for 5-min increments, beginning with 5 min and increasing to 40 min. For each item, participants indicated their confidence to execute the behavior on a 100-point percentage scale comprising 10-point increments, ranging from 0% (not at all confident) to 100% (highly confident). The internal consistency of this scale has been found to be excellent (alpha>.95) [36].

#### Disability

Disability was measured using the Late Life Function and Disability Instrument (LLFDI), which assesses disability in community-dwelling older adults [37]. The 16-item disability component has the participant rate activities, in terms of frequency and difficulty, for each item in this section (eg, How often do you participate in a given activity; to what extent do you feel limited in doing a particular activity?) The LLFDI limitation-scaled score ranges from 0-100 points. A score of 0 indicates no to low participation, whereas a score of 100 indicates high levels of participation in socially defined life tasks.

### Data Analysis

Feasibility, safety, and acceptability measures were analyzed using descriptive statistics. Mean changes were calculated for all secondary outcomes, and individual change scores were assessed to determine if they exceeded the minimal detectable change (MDC) or minimally clinically important difference (MCID), if known.

Data collected from peer coaches during the focus group and responses to open-ended questions in the satisfaction surveys were analyzed and coded for themes. Data from peer mentees’ open-ended questions within the satisfaction surveys were also analyzed and coded for themes. Coding for themes was completed by 1 researcher (CCS) with review by 2 additional researchers (TE and LQ).

## Results

### Feasibility: Recruitment Capability, Participation, and Retention

A total of 15 potential participants expressed interest in taking part in the study. In addition, 2 individuals did not agree to participate because they had pre-existing conflicts with the scheduled peer coach training sessions. Of the 13 that agreed to participate, 1 participant was excluded due to lower extremity peripheral neuropathy and the other due to cognitive impairment. This resulted in 11 participants enrolled in the study. One peer coach dropped out of the study due to time constraints. A total of 5 peer coaches completed the peer coach training. In summary, 10 individuals participated in the study, 5 peer coaches and 5 peer mentees (Figure 1). All individuals that finished the peer coach training (n=5) completed their roles as peer coaches over the 8-week intervention period. All peer mentees (n=5) completed the 8-week mentored walking program. All peer coaches and peer mentees exceeded the 80% criteria for retention. In all, 4 out of the 5 peer pairs completed 100% of weekly calls. Moreover, 1 peer pair missed 2 weekly calls due to scheduling conflicts. All peer dyads reached the 63% criteria for participation. A total of 4 out of the 5 peer pairs used the FitBit activity tracker and *friends* function without difficulty. Furthermore, 1 peer pair had technological difficulties (loss of the FitBit device, management of the battery, or syncing the activity tracker with a personal device).

### Resulting Sample Characteristics

The majority of the participants were male and highly educated. Participant characteristics are presented in Table 1.

**Table 1:** Demographics of participants.

Variable		Peer coach (n=5)	Peer mentee (n=5)
Age in years (SD)		64.6 (4.04)	63.4 (2.06)
Education in years (SD)		18.0 (0.89)	16.8 (1.02)
Male, n (%)		3 (60)	3 (60)
Race (white), n (%)		5 (100)	4 (80)
Disease duration in years (SD)		5.2 (1.24)	6.2 (2.2)
**Hoehn and Yahr stage, n (%)**			
	Stage 1	3	1
	Stage 2	1	3
	Stage 3	1	1

### Safety

No adverse events occurred over the duration of the study.

### Acceptability

All peer coaches (100%, 5/5) agreed that the material presented in the training was clear; however, some (40%, 2/5) reported difficulty with the length of the in-person training sessions and had suggested shorter sessions. The majority (80%, 4/5) of the peer coaches felt confident in their ability to be a peer coach after the training; however, 1 individual (20%) was neutral in their confidence to be a peer coach. All peer mentees (100%, 5/5) enjoyed interacting with their peer coaches. The majority (60%, 3/5) of peer mentees agreed that their peer coaches helped them to become confident to manage their walking activity; however, 2 (40%) of the peer mentees were neutral about the peer coach building their confidence. All participants (100%, 10/10) who participated in the peer interaction would recommend this peer coaching program to others with PD. All participants (100%, 10/10) were satisfied or very satisfied with the peer coach training or peer-mentored walking program.

### Participant Perspectives (Focus Group and Open-Ended Questions)

#### Peer Coach Training

Peer coaches recommended shorter in-person training sessions due to fatigue and difficulty learning new material all at one time. Peer coaches had a positive reaction to learning coaching skills, which included active listening, and being flexible and nonprescriptive. Coaches reported ease of use with the training manual and Web-based resources that were completed independently in the home environment.

#### Peer-Mentored Walking Program

Themes that emerged from peer coaches and peer mentees included factors that enhanced or deterred rapport or communication as well as factors that enhanced or deterred physical activity. Rapport and communication enhancers included sharing feelings, goals, and experiences. All peer coaches reported being able to successfully interact with their peer mentee via the mHealth platform. Some participants desired face-to-face interactions to enhance rapport. One example of this theme was a participant stating:

Starting with a face-to-face meeting establishes rapport and would be helpful.

Others indicated that they “would have liked to meet the person” and that they would have liked to “do some things together.” Rapport and communication deterrents included time constraints, power dynamics, and difficulties hearing over the phone. This theme, specifically power dynamics, was illustrated by 1 peer coach stating:

She [mentee] kept changing subjects and not answering questions that I [coach] asked.

Physical activity enhancers included competition and activity monitoring by peer pairs using the activity tracker and mHealth app. Peer coaches described the effect of sharing walking data via the mHealth platform as creating a “friendly competition,” a “gentle rivalry,” and “encouraging each other.”

One participant commented when asked about what was positive about the peer interaction:

...the competitive nature was even more motivating than just using the FitBit [alone].

Physical activity deterrents included time constraints with 1 peer coach stating:

I was going through a really busy time at work and so I was much less active.

The themes from the peer coaches and peer mentees were consistent between pairs and reflected what the opposite member reported.

### Walking Activity

**Figure 2:**
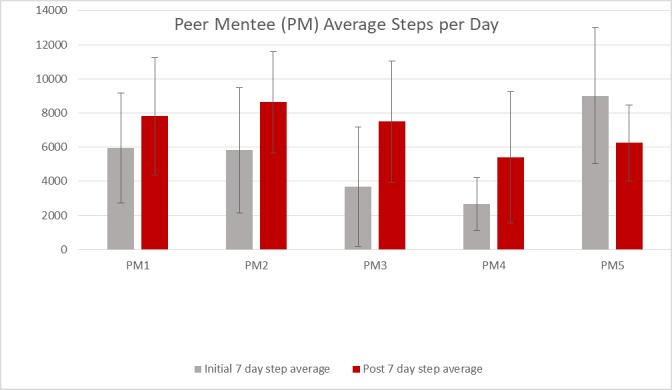
Peer mentees' initial and post average steps per day.

**Figure 3:**
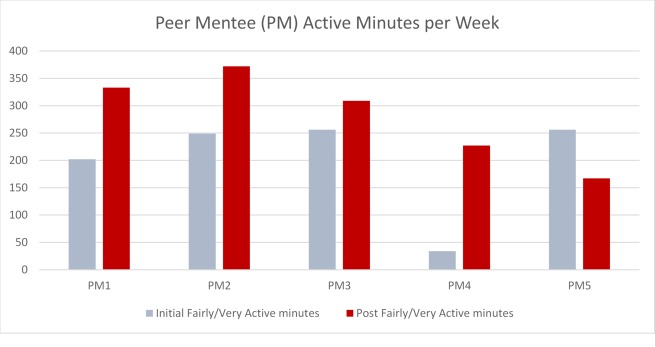
Peer mentees' initial and post fairly to very active minutes per week.

In the peer mentees, mean steps per day increased by 31% from 5428 (SD 2440) to 7115 (SD 1291) steps. The increase in 4 of the 5 peer mentees exceeded the MCID of 779 steps per day reported for individuals with a chronic neurological condition [38] (Figure 2). In all peer mentees, mean active minutes (fairly active to very active minutes) per week increased by 42% from 199 (SD 95) to 282 (SD 83) min per week. The change in these active minutes ranged from a decline of 89 min to an increase of 193 min per week, in the peer mentees (Figure 3). The MCID for active minutes in those with PD has yet to be determined. At baseline, peer mentees were achieving the recommended daily 30 min of fairly active to very active minutes 43% of the week (~3/7 days per week). After participating in the intervention, they were achieving this recommended activity level 63% of the week (~4/7 days per week).

### Self-Efficacy

The mean self-efficacy for peer mentees increased from 66.8 (SD 25.7) points at baseline to 70 (SD 25.9) points post intervention. Clinically important differences were not established for this measure.

### Disability

For the LLFDI, the mean score (limitation score) was 72.2 (SD 5.9) points at baseline and increased (improved) to 73.7 (SD 10.0) post intervention. A total of 3 of the 5 peer mentees had an increase or improvement (1.76-9.28 points); however, these changes did not exceed the MDC (MDC 90) of 11.62 points, suggesting that changes in disability were not clinically meaningful [37].

## Discussion

### Principal Findings

The purpose of this study was to develop and evaluate the feasibility, safety, and acceptability of a peer coach training program and a remote peer-mentored walking program to promote physical activity for persons with PD. In addition, we sought to examine preliminary evidence of individual-level changes in walking activity, self-efficacy, and disability in the peer mentees. This study revealed that people with PD could be successfully trained as coaches with the goal of increasing physical activity in peers with PD. The remote peer-mentored walking program was feasible with 4 out of the 5 peer pairs completing all 8 phone calls and most (4 out of the 5) peer coaches successfully using the mHealth platform to share walking data. The program was safe with no adverse events reported during the study period. The peer coach training program was acceptable with 100% of the coaches reporting being satisfied to very satisfied with the coaching program and feasible with 5 out of the 6 peer coaches completing the training program. Both programs were acceptable to peer coaches and peer mentees, with 100% recommending peer coach training/peer-mentored walking program to others with PD. Clinically meaningful gains in walking occurred in 4 out of the 5 peer mentees with low levels of physical activity (<6000 steps per day) at baseline, suggesting the potential benefits of a peer mentoring approach to improve physical activity in persons with mild to moderate PD who are physically inactive.

### Comparison With Prior Work

Peer coaches, in this study, were individuals with PD who were consistently walking briskly for greater than or equal to 150 min per week, based on the national physical activity guidelines [11,14]. However, it is unknown if the best person to be a coach is one who has already reached the targeted goal or one that is concurrently working on a target goal with the peer mentee. Sharing both stressful and rewarding experiences creates successful peer relations; therefore, the optimal walking physical activity criteria for a peer coach require further exploration [39]. In addition, research regarding the best method to match peer coaches and mentees is in its infancy [40,41]. Peer mentees were matched with a peer coach based on sex only, based on previous successful peer coaching interventions [20,22] and qualitative data indicating this preference [35]. However, peers expressed a desire for matching based on other potentially important characteristics (ie, career, education level, exercise mode, and geography). Other studies matched peers on sociocultural characteristics such as race [42,43] and revealed improvements in glucose control in those with diabetes [42] and decreased depressive symptoms in breast cancer survivors [43]. Different forms of peer matching have yet to be directly compared.

The majority (4 out of the 5) of peer mentees increased their steps per day (1864-3794 steps per day) and exceeded the MCID of 779 steps per day suggested for individuals with chronic progressive neurological disease [37]. Our finding of a 31% increase in mean steps per day among peer mentees is important because a 30% deficit has been reported in steps per day in people newly diagnosed with PD (compared with those without PD)[8]. The increase in active minutes per week is encouraging due to the large decline in active minutes per week (nearly 45 min per week) found in a previous 12-month observational study of walking activity in people with PD [7]. Finally, two of the peer mentees were no longer categorized as sedentary by week 8 [44]. Although gains in walking cannot be attributed to the peer mentoring program in this uncontrolled study, these results suggest the potential of this approach in persons with PD. Larger controlled trials in PD are needed to determine the effectiveness of this approach in increasing physical activity.

Although 4 out of the 5 peer mentees did experience increased daily step averages, there was not a commensurate decrease in disability. Given the relatively slow progression of PD that occurs over many years, it is likely that active engagement with increased levels of physical activity over longer periods would be necessary to reduce disability. In addition, the responsiveness of the LLFDI in PD is not known. Given that the intervention specifically targets walking, measures that focus on walking-related changes in disability (ie, 6-min walk test, 10-meter walk) may be more responsive and should be included in future studies.

We targeted self-efficacy through the FitBit *friends* feature and through motivational interviewing during phone conversations. Remote interactions between the peer coach and peer mentee provided an opportunity for goal setting and feedback on whether goals were attained. Peer coaches provided affirmations to increase empowerment and self-management of physical activity levels while living with PD. Mentees could see the coaches’ step counts, providing a social comparison and vicarious experiences that may have contributed to the positive but small increase in self-efficacy observed over the course of this study. Further investigation in a larger controlled trial is necessary to determine if self-efficacy is an important mediator of change in physical activity levels.

Although other studies have reported successful strategies to increase physical activity levels in persons with chronic neurological conditions, they rely on health care professionals to deliver the intervention. In a study that aimed to increase physical activity in individuals with multiple sclerosis, the behavioral coach was a graduate student with expertise in behavior change and physical activity [45]. A behavioral intervention delivered by physical therapists in persons with PD resulted in an increase in physical activity as measured by activity monitors [46]. Reliance on health care professionals may be cost-prohibitive for long-term application. A remote peer-mentoring approach using mHealth technology also allows for social modeling and shared experiences of living with the same condition, a potentially important element to facilitate meaningful lifestyle changes over the long term [27,47]. The use of remote peers, rather than health care professionals, offers a potentially cost-effective and scalable option to reduce sedentary behavior in those with PD and other chronic neurological conditions [48]. Embedding peer coaching within a preexisting health care structure (eg, partnering with physical therapists, neurologists, or movement disorder specialists) may optimize broader implementation and requires further investigation [49].

### Limitations

There are several limitations of this study. Considering the long-term nature of PD, the feasibility of peer coaching was examined over a relatively short period (8 weeks). The long-term feasibility of peer coaching in PD requires further investigation. In addition, this study did not have a control group; therefore, the increases in physical activity cannot be attributed to the peer coaching intervention. In addition, our sample was small, highly educated, and lacking in racial diversity; therefore, the results may not be generalizable to the broader population of people with PD. Selection bias may also limit generalizability of our results as participants were volunteers interested in participating in an exercise study. Although consumer-based activity trackers (FitBit) have been shown to be reasonably accurate in measuring step counts in the healthy population, the accuracy in those with PD has not been established [50]. Despite this limitation, waist-worn commercially available accelerometers are ecologically valid tools that are supported for use in clinical trials assessing walking activity in those with neurological conditions [51].

### Conclusions

Training people with PD to provide coaching targeting physical activity in persons with PD is a feasible approach. In addition, a remotely delivered peer-mentored walking program using mHealth technology is a feasible, safe, and acceptable approach in persons with mild to moderate PD. Larger controlled trials over longer periods are needed to further investigate the effect of peer coaching on increasing physical activity with the goal of improving function and reducing disability in those with PD.

## Abbreviations

LLFDI: Late Life Function and Disability Instrument
MCID: minimally clinically important difference
MDC: minimal detectable change
mHealth: mobile health
MOCA: Montreal Cognitive Assessment
PD: Parkinson disease
